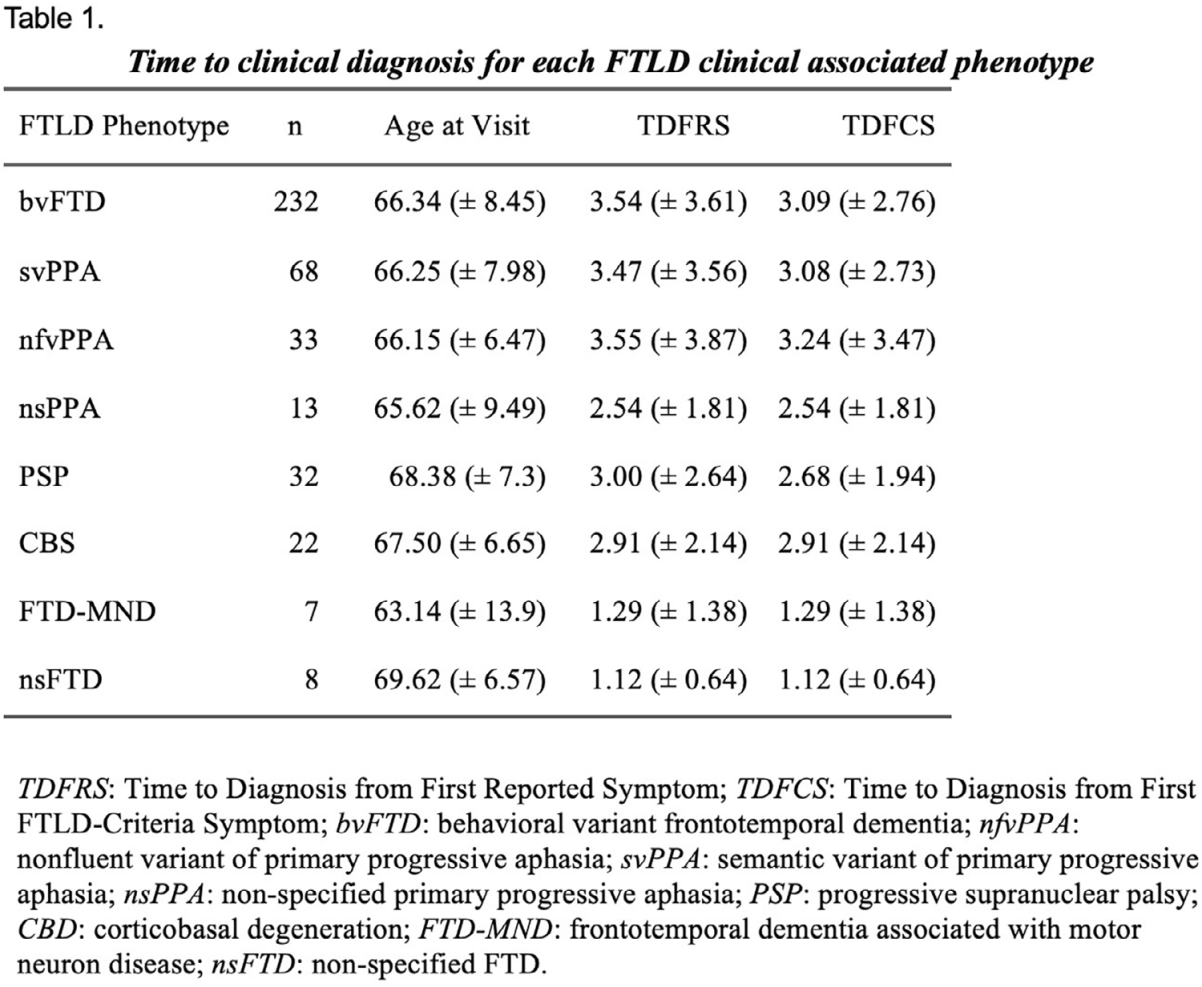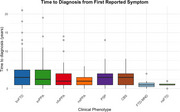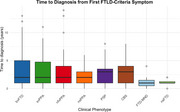# Time to diagnosis in FTLD clinical syndromes in Latin America: an analysis of the RedLat consortium

**DOI:** 10.1002/alz70857_106810

**Published:** 2025-12-26

**Authors:** Nahuel Magrath Guimet, Florentina Morello Garcia, Loana De Los Santos, Carolina Agata Ardohain Cristalli, Maria Eugenia Tabernero, Ricardo Allegri, Bruce L. Miller, Jennifer S. Yokoyama, Stefanie Pina‐Escudero, Katherine L. Possin, Diana L Matallana, Nilton Custodio, Andrea Slachevsky, Agustin Ibanez, José Alberto Avila Funes, Leonel Tadao Takada, Elisa de Paula, França Resende, María Isabel Behrens, David Aguillon, Kenneth S. Kosik, J. Nicholas Cochran, Victor Valcour

**Affiliations:** ^1^ Global Brain Health Institute, University of California, San Francisco, San Francisco, CA, USA; ^2^ Fleni, Buenos Aires, Buenos Aires, Argentina; ^3^ Institute of Neurosciences (INEU), Fleni‐CONICET, Buenos Aires, Buenos Aires, Argentina; ^4^ University of Buenos Aires, Buenos Aires, Buenos Aires, Argentina; ^5^ Santa Catalina Neurorehabilitación Clínica, Buenos Aires, Argentina; ^6^ Fleni, Buenos Aires, Argentina; ^7^ CONICET, Buenos Aires, Argentina; ^8^ Department of Neurology, Memory and Aging Center, University of California San Francisco, San Francisco, CA, USA; ^9^ Memory and Aging Center, Department of Neurology, Weill Institute for Neurosciences, University of California, San Francisco, San Francisco, CA, USA; ^10^ Global Brain Health Institute (GBHI), University of California San Francisco (UCSF); & Trinity College Dublin, San Francisco, CA, USA; ^11^ Latin American Brain Health Institute (BrainLat), Universidad Adolfo Ibáñez, Santiago, Región Metropolitana de Santiago, Chile; ^12^ University of California, San Francisco, San Francisco, CA, USA; ^13^ Global Brain Health Institute, San Francisco, CA, USA; ^14^ Memory and Aging Center, Weill Institute for Neurosciences, University of California, San Francisco, San Francisco, CA, USA; ^15^ Pontificia Universidad Javeriana, Bogota, Cundinamarca, Colombia; ^16^ Center for Memory and Cognition, Hospital Universitario San Ignacio Bogotá, Bogotá, Bogotá, Colombia; ^17^ Hospital Universitario San Ignacio, Bogotá, Bogotá, Colombia; ^18^ Unit Cognitive Impairment and Dementia Prevention, Peruvian Institute of Neurosciences, Lima, Peru, Lima, Lima, Peru; ^19^ Memory and Neuropsychiatric Clinic (CMYN), Neurology Service, Hospital del Salvador and Faculty of Medicine, Universidad de Chile, Santiago, Chile; ^20^ Geroscience Center for Brain Health and Metabolism (GERO), Santiago, Chile; ^21^ Global Brain Health Institute (GBHI), Trinity College Dublin (TCD), Dublin, Dublin, Ireland; ^22^ Latin American Brain Health Institute (BrainLat), Universidad Adolfo Ibañez, Santiago, Chile; ^23^ Instituto Nacional de Ciencias Médicas y Nutrición Salvador Zubirán, México, DF, Mexico; ^24^ Hospital das Clínicas, University of Sao Paulo Medical School, São Paulo, São Paulo, Brazil; ^25^ Cognitive and Behavioral Neurology Unit, Hospital das Clinicas HCFMUSP, Faculdade de Medicina, Universidade de Sao Paulo, Sao Paulo, Sao Paulo, Brazil; ^26^ Faculdade de Medicina de Ciências Médicas de Minas Gerais, Belo Horizonte, Brazil; ^27^ Hospital Clínico de la Universidad de Chile, Santiago de Chile, Chile; ^28^ Neurosciences Group of Antioquia, University of Antioquia, Medellín, Colombia; ^29^ University of California Santa Barbara, Santa Barbara, CA, USA; ^30^ HudsonAlpha Institute for Biotechnology, Huntsville, AL, USA; ^31^ Memory and Aging Center, University of California San Francisco, San Francisco, CA, USA

## Abstract

**Background:**

Frontotemporal lobar degeneration (FTLD) includes behavioral variant frontotemporal dementia (bvFTD), nonfluent and semantic variants of primary progressive aphasia (nfvPPA, svPPA), progressive supranuclear palsy (PSP), corticobasal degeneration (CBD), and frontotemporal dementia associated with motor neuron disease (FTD‐MND). These syndromes are associated with prolonged time to diagnosis, impacting patient care and family outcomes. While diagnostic timelines have been studied in other regions, data from Latin America remain limited. Addressing this gap is essential to identify barriers and improve timeliness of diagnosis and treatment strategies.

**Method:**

We analyzed 415 cases (mean age 66.5 ± 8.2 years) from the RedLat consortium (2021–2024): bvFTD (*n* =  232, 55.9%), svPPA (*n* =  68, 16.4%), nfvPPA (*n* =  33, 8%), non‐specified PPA (nsPPA; *n* =  13, 3.1%), PSP (*n* =  32, 7.7%), CBS (*n* =  22, 5.3%), FTD‐MND (*n* =  7, 1.7%), and non‐specified FTD (*n* =  8, 1.9%). Time to diagnosis was defined as the interval from first reported symptom (TDFRS) and first FTLD‐criteria symptom (TDFCS) to diagnosis. Clinical and sociodemographic factors influencing these timelines were also assessed.

**Result:**

Diagnostic timelines varied substantially. The most prevalent phenotype, bvFTD, had a mean (±SD) TDFRS of 3.54 ± 3.61 years and TDFCS of 3.09 ± 2.76 years. In contrast, svPPA (TDFRS = 3.47 ± 3.56; TDFCS = 3.08 ± 2.73) and PSP (TDFRS = 3.00 ± 2.64; TDFCS = 2.68 ± 1.94) showed similar durations. FTD‐MND exhibited the shortest timeline, with a mean duration of 1.29 ± 1.38 years for both TDFRS and TDFCS. Younger age at onset and lower education levels were associated with longer diagnostic timelines.

**Conclusion:**

This study highlights substantial variability in diagnostic timelines across FTLD phenotypes in Latin America. The longest timelines were seen for bvFTD, while FTD‐MND had the shortest. Younger patients and those with lower education faced greater challenges in obtaining timely diagnoses, suggesting sociodemographic disparities. These findings emphasize the need for targeted interventions, including increased clinical awareness, improved access to specialized care, and strategies to address disparities, optimizing the diagnostic process and outcomes for patients and families.